# Urban stormwater runoff negatively impacts lateral line development in larval zebrafish and salmon embryos

**DOI:** 10.1038/s41598-018-21209-z

**Published:** 2018-02-12

**Authors:** Alexander Young, Valentin Kochenkov, Jenifer K. McIntyre, John D. Stark, Allison B. Coffin

**Affiliations:** 1 College of Arts and Sciences, Washington State University, Vancouver, WA USA; 2 Puyallup Research and Extension Center, Washington State University, Puyallup, WA USA; 3 Department of Integrative Physiology and Neuroscience, Washington State University, Vancouver, WA USA; 4 Present Address: Arizona College of Osteopathic Medicine, Arizona, USA

## Abstract

After a storm, water often runs off of impervious urban surfaces directly into aquatic ecosystems. This stormwater runoff is a cocktail of toxicants that have serious effects on the ecological integrity of aquatic habitats. Zebrafish that develop in stormwater runoff suffer from cardiovascular toxicity and impaired growth, but the effects of stormwater on fish sensory systems are not understood. Our study investigated the effect of stormwater on hair cells of the lateral line in larval zebrafish and coho salmon. Our results showed that although toxicants in stormwater did not kill zebrafish hair cells, these cells did experience damage. Zebrafish developing in stormwater also experienced impaired growth, fewer neuromasts in the lateral line, and fewer hair cells per neuromast. A similar reduction in neuromast number was observed in coho salmon reared in stormwater. Bioretention treatment, intended to filter out harmful constituents of stormwater, rescued the lateral line defects in zebrafish but not in coho salmon, suggesting that not all of the harmful constituents were removed by the filtration media and that salmonids are particularly sensitive to aquatic toxicants. Collectively, these data demonstrate that sub-lethal exposure to stormwater runoff negatively impacts a fish sensory system, which may have consequences for organismal fitness.

## Introduction

After storm events in urban areas, water runs off from roads, bridges, and other impervious surfaces. Some of this runoff is conveyed to the sanitary sewer where it may be treated in a wastewater plant before being released into a waterway, however, most stormwater runoff is conveyed directly into aquatic environments^1^. This runoff may contain many chemicals deposited on road surfaces by vehicles, including metals and polycyclic aromatic hydrocarbons (PAHs) such as petroleum products, tire dust, exhaust, and brake pad dust^2–5^. Stormwater runoff represents a large input of non-point source pollution to local watersheds^4,6^. Pollutant concentrations vary widely between storm events and within a storm, with water from the earliest part of the storm (first flush) often containing the highest levels of many potential toxicants^3,4^. First flush water is therefore associated with the majority of the toxicity^7^. In addition, PAH mixtures are themselves complex and highly variable, making stormwater chemistry exceedingly complicated to decipher^8^.

Pollutants in stormwater runoff can negatively impact the development of aquatic life. Multiple studies demonstrate that stormwater can be fatal to larval fishes, including zebrafish and Pacific salmonids, as well as to juvenile and adult coho salmon^5,9–13^. Outside of acutely lethal effects, exposure to aquatic pollutants can cause sub-lethal effects that have long-term repercussions on organismal fitness^14,15^. In zebrafish and walleye embryos, stormwater or PAH exposure causes delayed development, cardiovascular dysfunction, and craniofacial deformities^9,12,13,16^. Cardiac defects are also reported in larvae of large marine fishes that contact crude oil, suggesting that cardiotoxicity is widespread in fish exposed to PAHs^17,18^. Early life exposure to PAHs can have long-lasting results, including negative impacts on cardiac structure and function in adulthood^19^. PAH exposure also alters neurodevelopment, and changes in locomotion were reported in adult fishes, suggesting delayed effects of embryonic PAH exposure on nervous system or muscle development^20,21^.

We examined the effects of urban stormwater runoff on the lateral line, the mechanosensory system on the head and body of fishes and aquatic amphibians. The lateral line is composed of stereotypically located clusters of cells, called neuromasts, that each contain sensory hair cells interdigitated with non-sensory supporting cells^22,23^. Fishes use this system to detect nearfield water movement from both abiotic and biotic sources, including predators, prey, and stream flow^24^. The lateral line contributes to behaviors such as orientation to current (rheotaxis), which may be important for navigation and migration^25,26^. As a surface structure, the lateral line directly contacts compounds in the aquatic environment, and sensory hair cells are highly susceptible to many toxicants of both biomedical and environmental origins^27–29^. The lateral line in larval zebrafish is an excellent model system for toxicology studies, owing to the small size of the organism, rapid *ex utero* development, and high fecundity^30^. The zebrafish lateral line is a tractable model system for toxicology studies, contributing to our understanding of how aquatic pollutants such as copper and bisphenol-A affect fish sensory systems^29,31–33^.

Here we assessed the effects of acute urban stormwater exposure on the lateral line in larval zebrafish. We also examined developmental effects of stormwater exposure in both zebrafish and coho salmon. Finally, we examined the effectiveness of green stormwater infrastructure, specifically soil bioretention treatments, to reduce stormwater-related lateral line defects. We found that acute stormwater exposure caused a reduction in hair cell viability, and that fish reared in stormwater had fewer hair cells per neuromast, and often fewer neuromasts, than fish raised in clean water. These data suggest that sub-lethal stormwater exposure can negatively impact sensory function, potentially impairing prey capture, predator avoidance, and rheotaxis.

## Results

### Acute exposure in older zebrafish larvae

**Figure 1:**
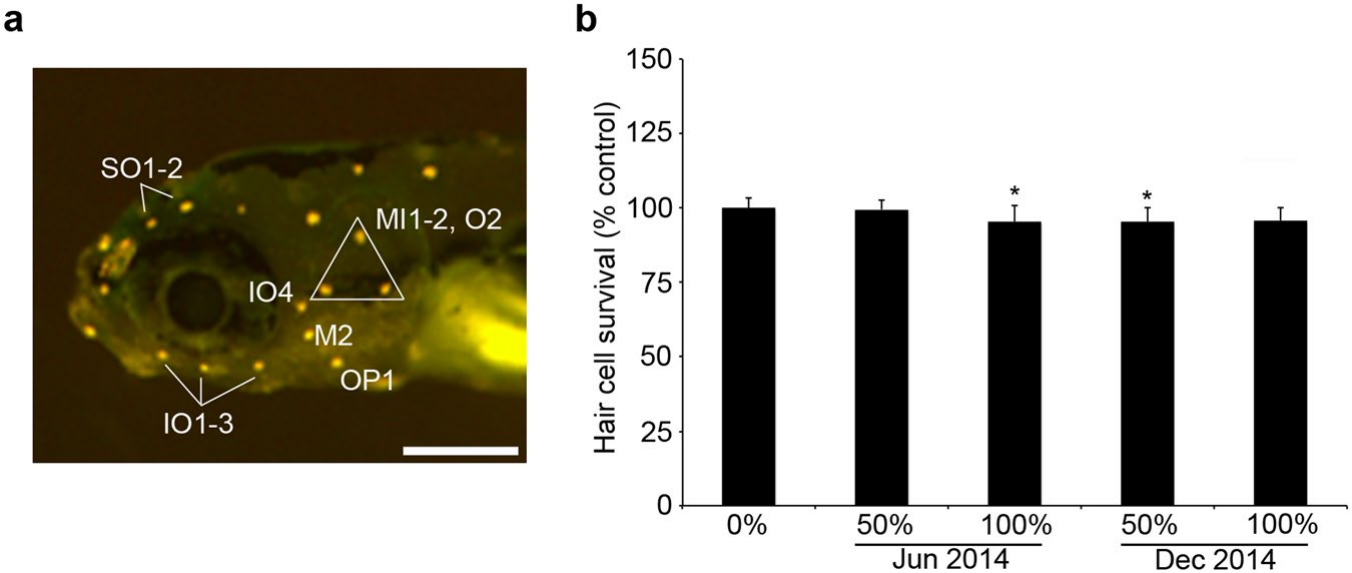
(**a**) Morphology of the head neuromasts in a larval zebrafish, visualized with DASPEI. Labels indicate the neuromasts that were quantified to assess hair cell damage. Scale bar = 250 µm. (**b**) Acute stormwater exposure does not kill lateral line hair cells. 5–6 dpf fish were exposed to stormwater for 24 hr (from June or December 2014 storm events), then hair cells were labeled with DASPEI. There is a slight but significant effect of stormwater (one-way ANOVA, F_4,62_ = 4.02, p = 0.006). Bonferroni-corrected post-hoc testing shows decreased hair cell survival for fish in the 100% June or 50% December groups (*p < 0.0001 for each). N = 13–14 fish per group, bars are + 1 s.d.

**Figure 2:**
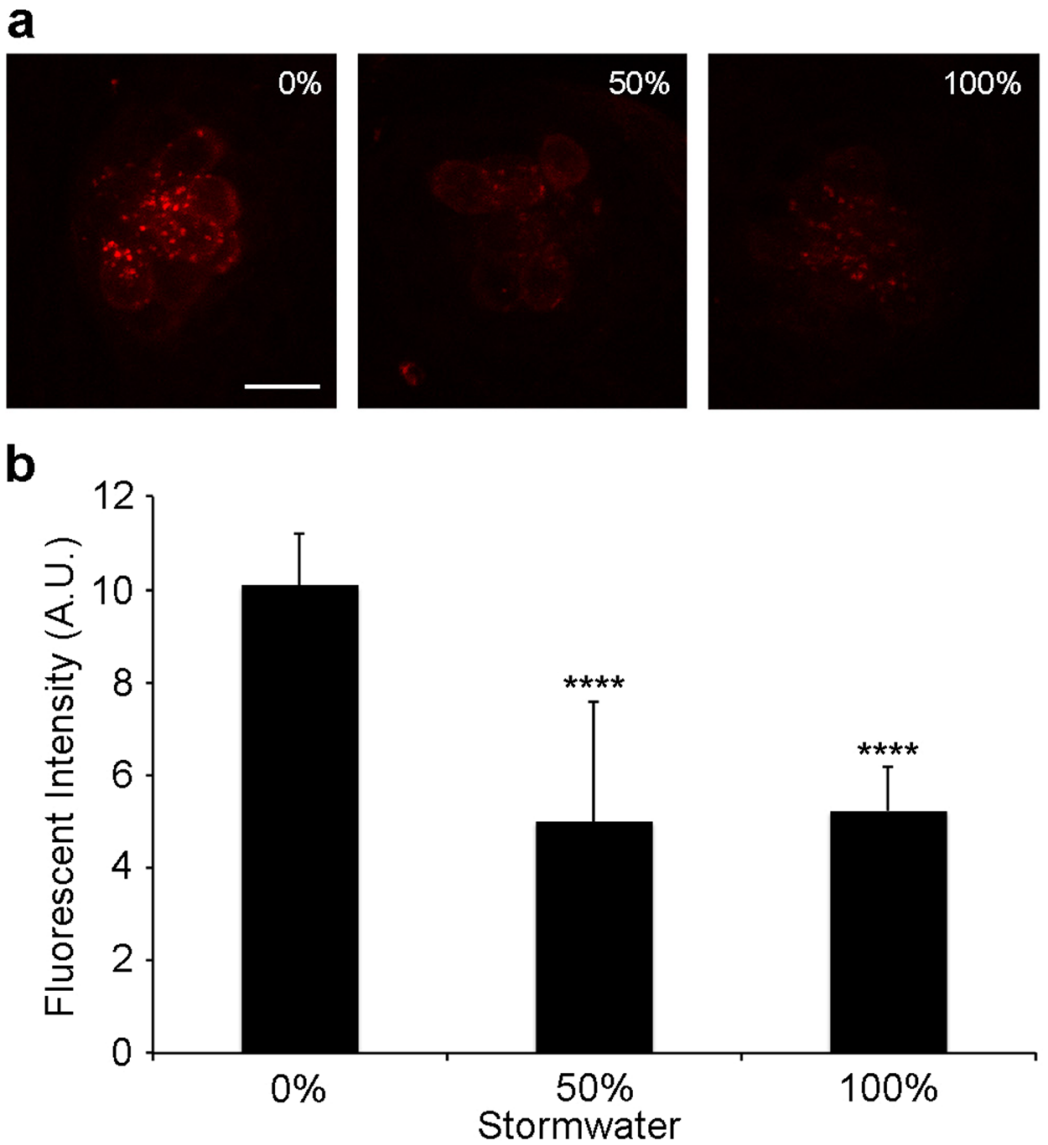
Acute stormwater exposure decreases uptake of the transduction-dependent dye FM 1–43FX by hair cells. (**a**) Representative confocal images of neuromast IO3 from fish treated with no stormwater (0%), 50%, or 100% stormwater for 24 hr, then labeled with FM 1–43FX. The bar in the left panel = 10 µm and applies to all panels. (**b**) There is a significant reduction in FM 1–43FX fluorescence in stormwater-treated fish (one-way ANOVA, F_2,25_ = 19.81, p < 0.0001). Bonferroni-corrected post-hoc testing shows decreased fluorescence at 50% or 100% stormwater (****p < 0.0001 for each). Fluorescence was quantified in arbitrary units (A.U.). This experiment used stormwater from a June 2014 storm event. N = 7–11 fish per group, bars are + 1 s.d.

We first exposed 5–6 days post-fertilization (dpf) larvae to a brief stormwater pulse to determine if short-term stormwater exposure was toxic to lateral line hair cells. Twenty-four hr of exposure to 50% or 100% stormwater did not kill lateral line hair cells (compared to control fish treated with embryo medium), as determined with quantification of DASPEI fluorescence or direct counts of GFP + hair cells in Brn3c:mGFP larvae (Fig. 1 and data not shown). We observed this same result with water from two different storm events, making it likely that runoff from other storms would have little effect on acute hair cell survival. However, water from either the December 2014 or June 2015 storms significantly reduced hair cell uptake of the vital dye FM 1–43FX (Fig. 2; F_2,25_ = 19.81, p < 0.0001), with approximately 50% reduction in FM 1–43FX fluorescence in all treatment groups. FM1–43FX uptake is a strong indicator of mechanotransduction channel function^34,35^. These results suggest that developed hair cells survive acute stormwater exposure but that function is compromised.

Zebrafish robustly regenerate damaged hair cells, and toxicants such as copper and bisphenol-A negatively impact regenerative potential^29,36^. We therefore examined hair cell regeneration during exposure to stormwater. Fish were treated with the hair cell toxicant neomycin^28,37^, then the neomycin was removed and fish were allowed to recover in stormwater (or fresh embryo medium as a control) prior to hair cell assessment. Stormwater exposure (50% or 100%) did not attenuate hair cell regeneration at the 24 or 48-hr timepoints (data not shown). These data suggesting that stormwater does not influence progenitor cell proliferation in relatively mature neuromasts, as hair cell regeneration after neomycin is almost entirely dependent on proliferation of neighboring supporting cells^36,38^.

### Stormwater exposure during zebrafish development

**Figure 3:**
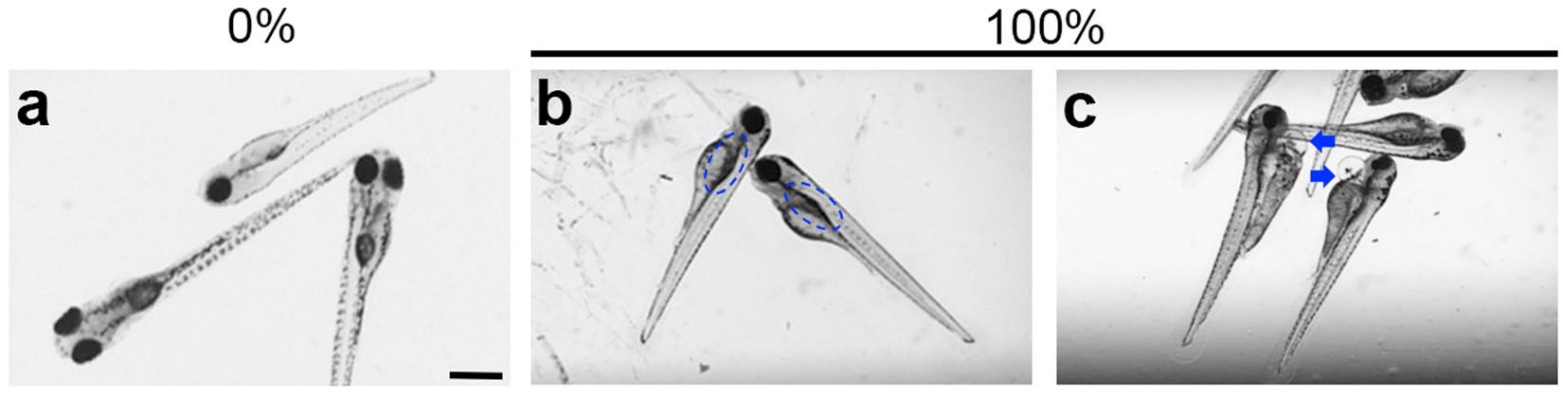
Stormwater alters gross morphology of 4 dpf larvae exposed to runoff throughout development. (**a**) Untreated larvae (0% stormwater) showing normal development. (**b**-**c**) Examples of stormwater-treated larvae. (**b**) Some treated fish have not yet inflated their swim bladders (blue outlines). (**c**) Other treated fish show pericardial edema (blue arrows). Scale bar in a = 0.5 mm and applies to all panels.

We then asked if exposure to stormwater runoff influenced lateral line development in zebrafish embryos. Fish reared in stormwater from 4 hpf to 4 dpf showed a variety of developmental phenotypes, ranging from normal gross morphology to cardiac edema and reduced swim bladder inflation, consistent with prior studies^5,13^ (Fig. 3). Stormwater also caused increased fish mortality, with up to 50% mortality observed for some stormwater concentrations from the January 2016 storm event. By contrast, no mortality was seen for the lower stormwater concentrations used from the June 2015 storm event. Control fish, which were raised in fish facility water, did not show gross morphological defects (Fig. 3a).

**Figure 4:**
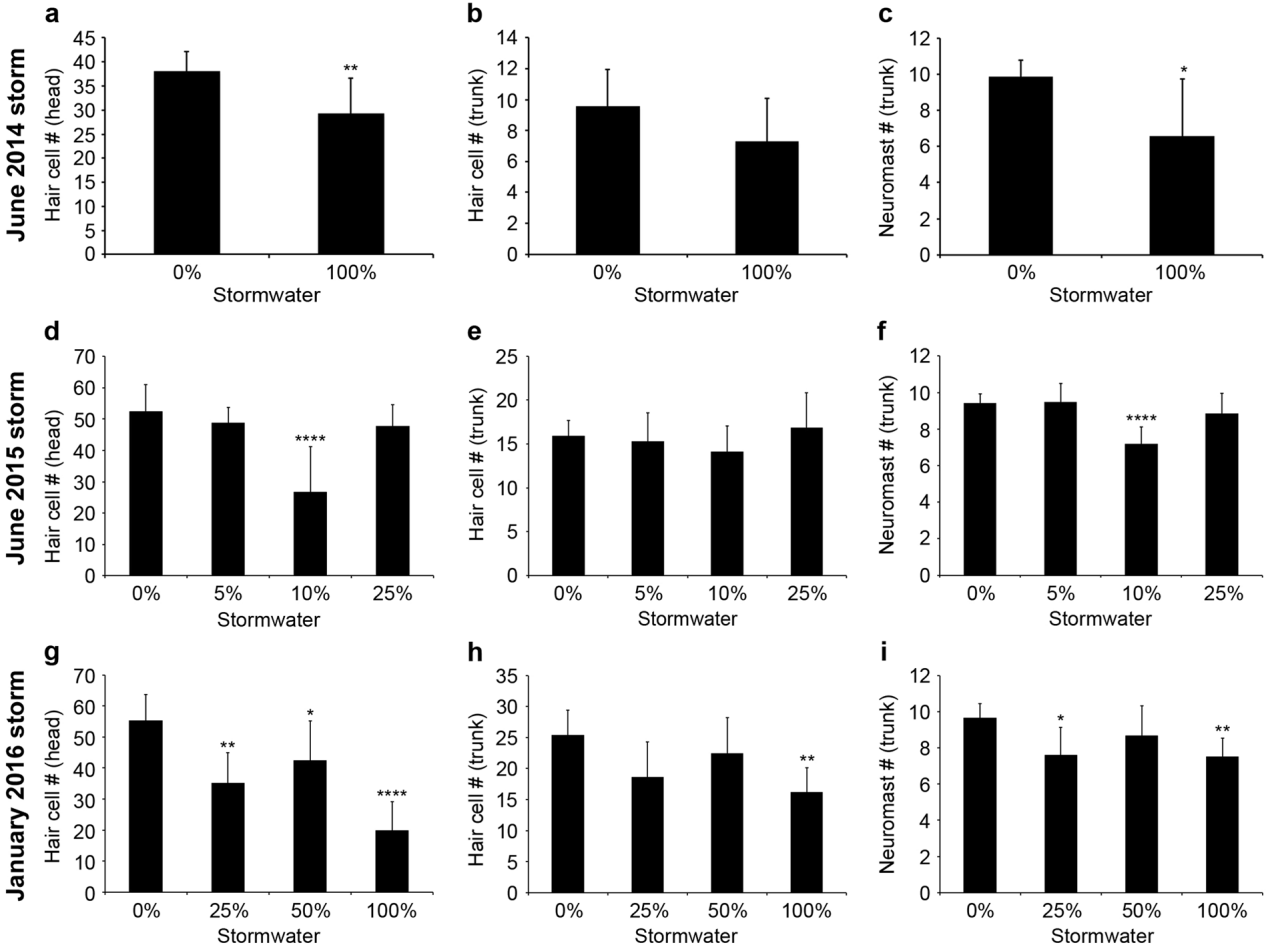
Stormwater runoff causes detrimental effects on zebrafish lateral line development. (**a**–**c**) show data from a storm in June 2014, (**d**–**f**) from a June 2015 storm, and (**g**–**i**) from a January 2016 storm. All experiments were performed on Brn3c:mGFP fish treated from 4 hpf to 4 dpf. (**a**–**c**) Fish treated in stormwater from June 2014 had (**a**) a significant decrease in hair cell number in five head neuromasts (2-tailed t-test, p = 0.009), (**b**) no significant difference in hair cell number in the two most terminal neuromasts on the tail (2-tailed t-test, p = 0.1024), and (**c**) a significant decrease in the number of trunk neuromasts (2-tailed t-test, p = 0.014). (**d**) Fish exposed to stormwater from June 2015 showed a significant decrease in hair cell number in head neuromasts (one-way ANOVA, F_3,43_ = 17.99, p < 0.0001). For this storm, concentrations higher than 25% were lethal to the fish. (**e**) The same stormwater did not have any significant effect on hair cell number in the two most anterior trunk neuromasts (one-way ANOVA, F_3,43_ = 1.688, p = 0.1837). (**f**) The number of trunk neuromasts was significantly decreased in fish treated with stormwater from June 2015 (one-way ANOVA, F_3,43_ = 15.84, p = 0.004). (**g**-**h**) Fish exposed to stormwater from January 2016 showed a significant decrease in hair cell number in head neuromasts (**g**; one-way ANOVA, F_3,27_ = 15.29, p < 0.0001), and trunk neuromasts (**h**; one-way ANOVA, F_3,28_ = 5.613, p = 0.0038). (**i**) There was also a significant decrease in the number of trunk neuromasts in the January 2016 group (one-way ANOVA, F_3,28_ = 5.564, p = 0.004). For all groups asterisks denote significant differences from untreated animals, as determined with t-tests (panels a–c) or Bonferroni-corrected posthoc testing (panels d–i) (*p < 0.05, **p < 0.01, ****p < 0.0001). N = 7–13 fish per group treatment, bars are + 1 s.d.

We consistently observed detrimental effects of stormwater exposure on lateral line development, but the magnitude of the effect differed based on the stormwater event (Fig. 4). Brn3c:mGFP fish treated with stormwater from 4 hpf – 4 dpf had fewer GFP + hair cells per neuromast (as compared to control animals reared in fish facility water), particularly for head neuromasts, and fewer neuromasts along the trunk. By contrast, treatment from 1–3 dpf had little effect on lateral line morphology (data not shown). Initial experiments with stormwater from June 2014 found that rearing in 100% stormwater resulted in a 23% reduction in hair cells in both head and trunk neuromasts, although only the reduction in head neuromasts was statistically significant (Fig. 4a,b; p = 0.009). These fish also had 33% fewer trunk neuromats overall (Fig. 4c). For the June 2015 stormwater event, we exposed the developing zebrafish to a low percentage of stormwater (5–25%) because high concentrations were lethal, suggesting a higher contaminant load in the June 2015 runoff. These lower stormwater concentrations caused a significant reduction in hair cells of head neuromasts (40%; F_3,43_ = 17.99, p < 0.0001) and in the number of trunk neuromasts (24% reduction; F_3,43_ = 15.84, p = 0.004), but only at the 10% stormwater concentration, an effect observed multiple times with water from this storm. Water from the January 2016 storm also caused a reduction in the number of hair cells per head neuromast for all three stormwater concentrations tested (25, 50, 100%), with decreases of 36%, 23%, and 64%, respectively (F_3,27_ = 15.29, p < 0.0001). There was also a significant reduction in hair cell number of anterior trunk neuromasts (in 100% stormwater only, 36% decrease; F_3,28_ = 5.613, p = 0.0038), and a reduction in the number of trunk neuromasts, with 21% fewer neuromasts in the 25% stormwater group, and 22% fewer neuromasts in the 100% stormwater group (Fig. 4h,i; F_3,28_ = 5.564, p = 0.004). Collectively, these data demonstrate that embryonic stormwater exposure causes lateral line defects in zebrafish larvae.

**Figure 5:**
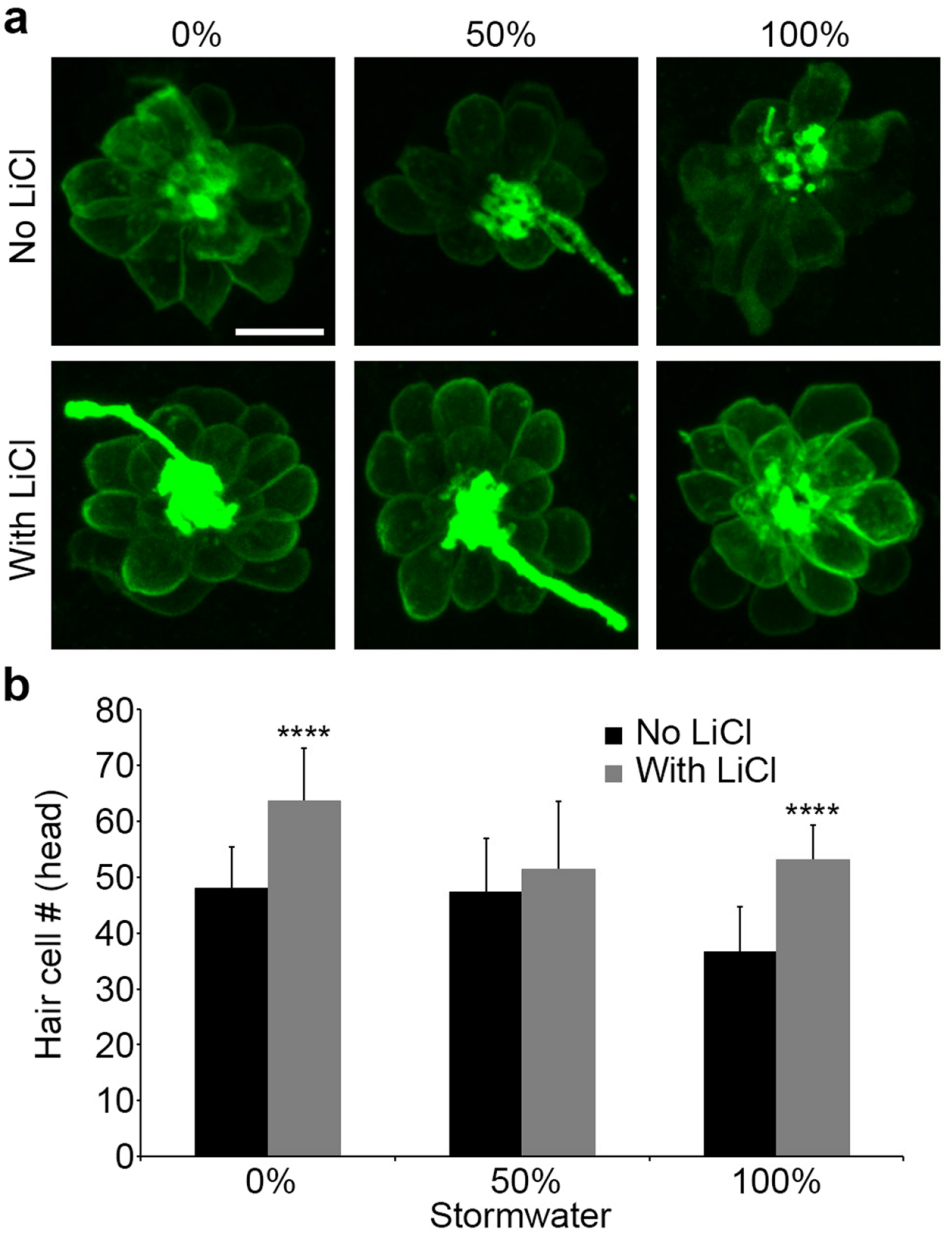
The Wnt activator Lithium chloride (LiCl) reduces the detrimental effects of stormwater treatment on the developing lateral line. All experiments were performed on Brn3c:mGFP fish treated from 4 hpf to 4 dpf. (**a**) Representative confocal images of the OP1 neuromast from zebrafish larvae raised in different stormwater concentrations, with and without the presence of LiCl. The scale bar in the upper left = 10 µm and applies to all panels. (**b**) Quantitative data showing significantly fewer hair cells in fish raised in stormwater from January 2016 (black bars), and prevention of hair cell loss when fish were raised in the same stormwater with LiCl present (gray bars). Similar to previous experiments, stormwater caused a significant decrease in hair cell number (two-way ANOVA, F_2,72_ = 10.15, p = 0.0001). LiCl-treated fish had significantly more hair cells than fish reared without LiCl (two-way ANOVA, F_1,72_ = 35.09, p < 0.0001). The interaction between the stormwater and LiCl treatments was also significant (two-way ANOVA, F_2,72_ = 3.819, p = 0.026). Asterisks indicate significant pairwise differences between -LiCl and + LiCl groups for a given stormwater concentration (****p < 0.0001). N = 10–16 fish per group treatment, bars are + 1 s.d.

We next examined possible causes of the lateral line defect. We focused on Wnt signaling, as this pathway is critical for neuromast deposition, cell proliferation, and hair cell addition in deposited neuromasts^39–42^. Zebrafish were treated with 50% or 100% stormwater from the January 2016 storm event in the presence or absence of the Wnt activator LiCl (15 mM). We elected to focus on the head neuromasts, as all storm events led to a significant reduction in hair cell number in this region. As previously found, 100% stormwater exposure during development reduced the number of hair cells in the head neuromasts (compared to facility water-treated controls), and this effect was rescued by co-treatment with LiCl (Fig. 5; p < 0.0001). LiCl treatment also significantly increased the number of hair cells in fish treated with facility water only (no stormwater), consistent with prior reports^41,42^.

**Figure 6:**
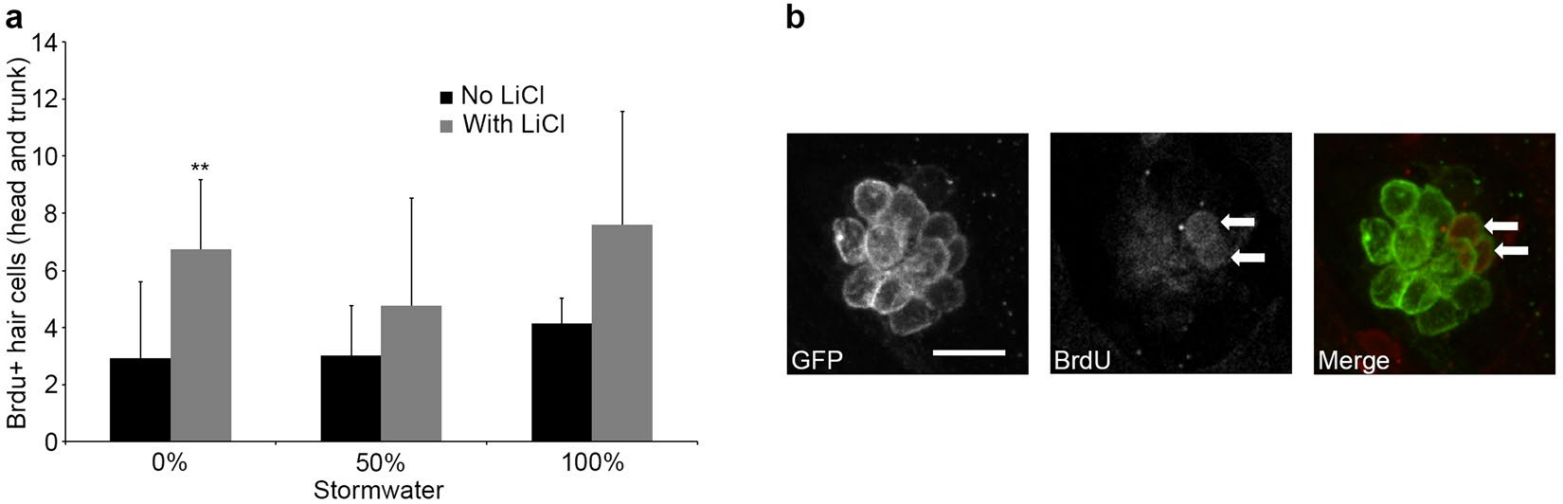
Developmental stormwater exposure does not alter cell proliferation within zebrafish neuromasts. Experiments were performed in Brn3c:mGFP fish, allowing for quantification of BrdU + /GFP + cells (hair cells). Fish were exposed to stormwater from 4 hpf – 4 dpf, and BrdU was pulsed at 32.5–34 hpf. (**a**) There was no change in the number of BrdU + hair cells in stormwater-treated embryos (black bars, two-way ANOVA, F_2,44_ = 1.701, p = 0.19). There was a significant increase in BrdU-labeled hair cells in LiCl-treated zebrafish (gray bars, two-way ANOVA, F_1,44_ = 13.72, p = 0.0006). Asterisks indicate significant pairwise differences between -LiCl and + LiCl groups for a given stormwater concentration. N = 7–13 zebrafish per group with exception of 50% stormwater + LiCl, where N = 4. Bars are + 1 s.d. (**b**) Representative confocal images showing BrdU labeling in the P2 neuromast of the trunk, with two hair cells double-labeled with GFP and BrdU (white arrows). Scale bar = 10 µm and applies to all panels.

Since Wnt signaling regulates cell division in the lateral line, we next examined cell proliferation with a BrdU assay in Brn3c:mGFP larvae. BrdU + hair cells were defined as those cells which were also GFP+. There was no significant difference in the number of BrdU-labeled hair cells in stormwater-treated fish compared to controls reared in fish facility water (2.9–4.1 BrdU+/GFP + hair cells across groups), suggesting that stormwater does not negatively impact cell proliferation in the developing lateral line (Fig. 6). However, there was a significant increase in BrdU-labeled hair cells in fish treated with LiCl, regardless of stormwater treatment (F_1,44_ = 13.72, p = 0.0006), consistent with previous studies showing that Wnt activation stimulates proliferation during lateral line development^42^. The combination of stormwater, LiCl, and BrdU was particularly toxic to zebrafish embryos, with 70–80% mortality observed in these groups.

**Figure 7:**
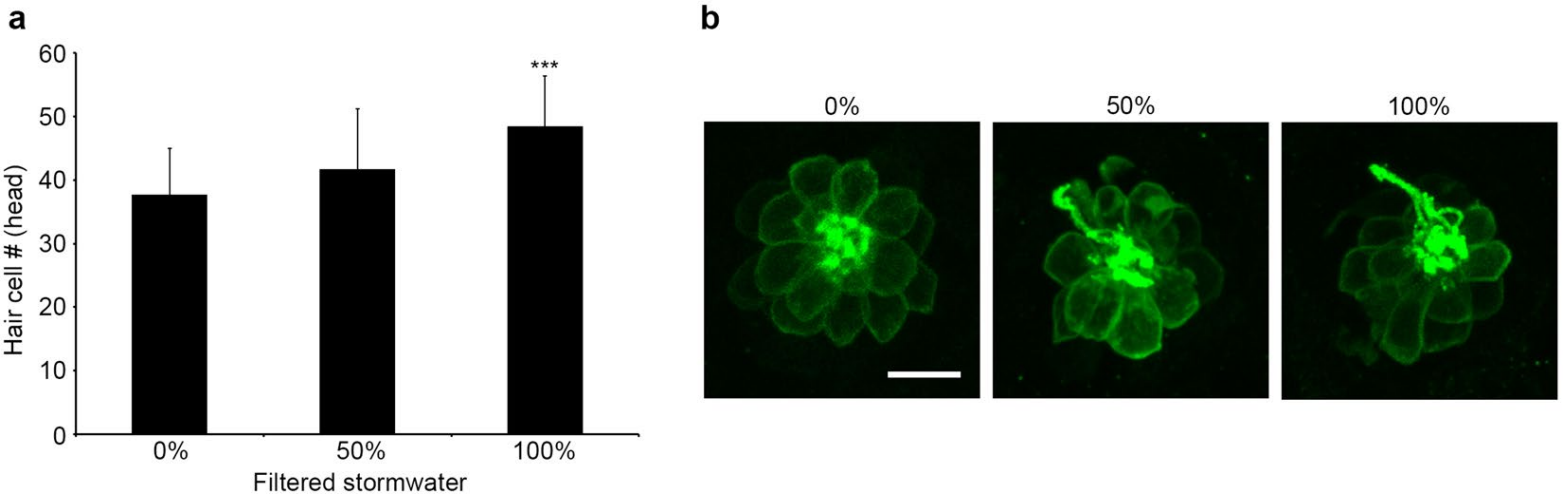
Filtered stormwater rescues neuromasts from the toxic effects of stormwater. All experiments were performed on Brn3c:mGFP fish treated from 4 hpf to 4 dpf. Filtered stormwater was diluted with EM; 0% therefore represents fish treated with clean water. (**a**) Fish exposed to filtered stormwater from January 2016 showed a significant increase in hair cell number in head neuromasts (one-way ANOVA, F_2,63_ = 8.092, p = 0.0007); post-hoc testing (Bonferroni-corrected) shows increase in the 100% filtered stormwater group (***p < 0.001). (**b**) Representative confocal images of the OP1 neuromast from zebrafish larvae raised in different filtered stormwater concentrations. N = 19–24 fish per group treatment, bars are + 1 s.d., scale bar = 10 µm and applies to all panels in (**b**).

Previous research showed that stormwater filtration with bioretention columns significantly reduced developmental toxicity in zebrafish embryos^5,11,13^. We therefore asked if filtration attenuated the lateral line defects caused by stormwater exposure. As shown in Fig. 7, larvae raised in filtered stormwater had more hair cells than larvae reared in clean fish facility water, with 29% more hair cells observed in fish treated with 100% filtered stormwater compared with clean water controls (F_2,63_ = 8.092, p = 0.0007). Data are shown for the head neuromasts only, as the most consistent effect of stormwater rearing was observed in this region. These data suggest that stormwater treatment with bioretention columns is sufficient to remove the toxicants that affect lateral line development. There was no increase in mortality in fish reared in filtered stormwater vs. clean fish facility water.

### Effects of stormwater on coho lateral line development

**Figure 8:**
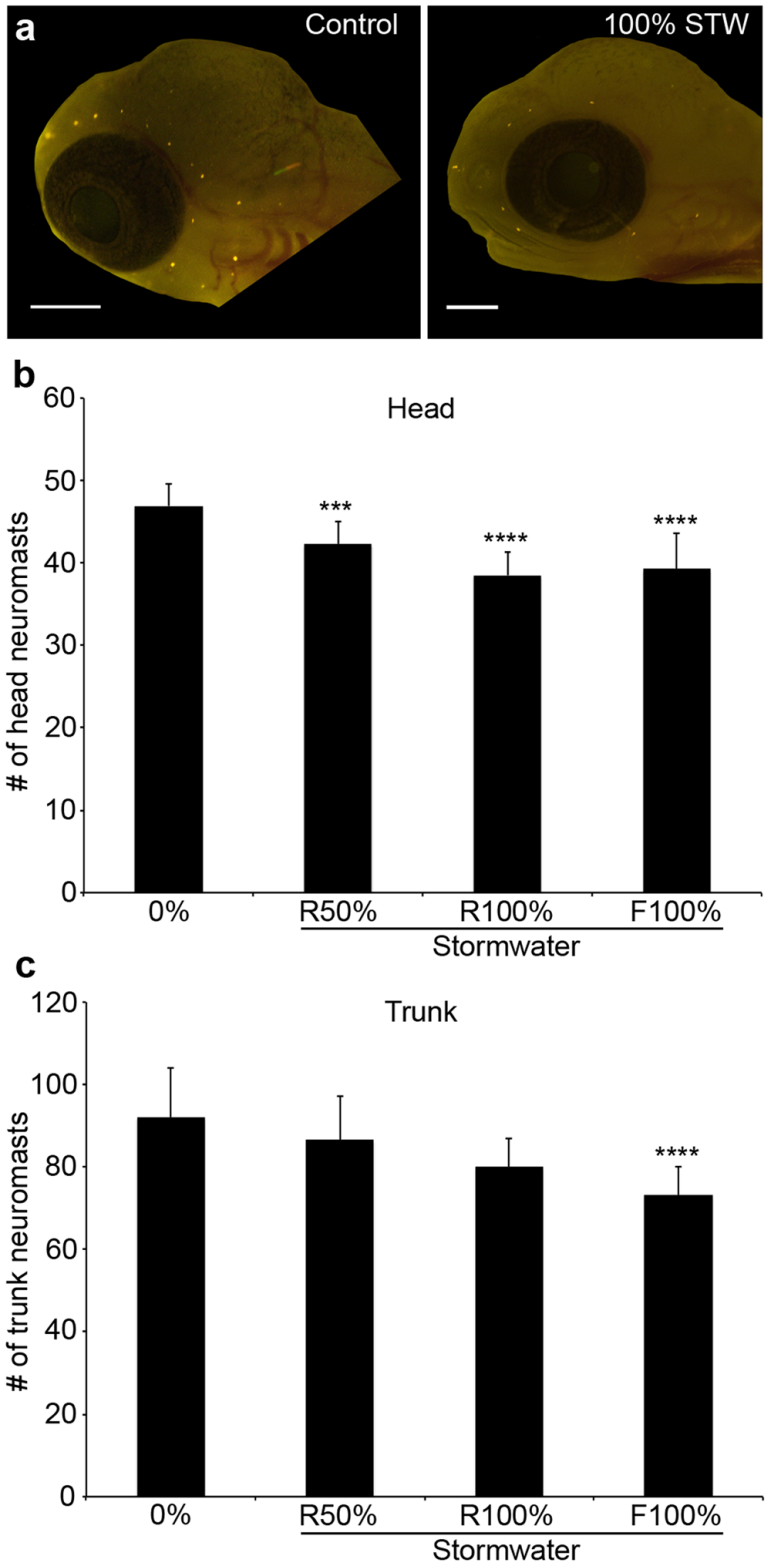
Stormwater exposure during development reduces the number of lateral line neuromasts in coho salmon. (**a**) Fluorescent images of DASPEI-labeled coho salmon embryos from a control fish (left) and a fish exposed to 100% stormwater (STW; right). Scale bars = 0.5 mm. (**b**) There are fewer head neuromasts in stormwater-exposed embryos (one-way ANOVA, F_3,46_ = 14.84, p < 0.0001). R = regular (unfiltered) stormwater, F = filtered stormwater. (**c**) There are also fewer trunk neuromasts in these embryos (one-way ANOVA, F_3,46_ = 8.33, p = 0.0002). For both (**b**) and (**c**), Bonferroni-corrected post-hoc analyses demonstrate significant differences from control embryos (***p < 0.001, ****p < 0.0001). N = 6–20 fish per treatment, bars are + 1 s.d.

While zebrafish are a tractable model for toxicity studies, in the Pacific northwest, Pacific salmonids (*Oncorynchus* spp.) are of greater ecological and economic interest. We therefore used coho salmon to better understand how stormwater influences lateral line development in a commercially important species. Coho salmon embryos were episodically exposed during rearing to 50% stormwater diluted in well water, 100% stormwater, or 100% stormwater that was first filtered through a bioretention column. Coho embryos treated with any of these three treatments had fewer head neuromasts than control embryos continuously reared in well water, with a 10% reduction in head neuromasts in embryos treated with 50% stormwater, and an 18% reduction in neuromasts in embryos treated with 100% stormwater or filtered stormwater (Fig. 8a,b; F_3,46_ = 14.84, p < 0.0001). There was also an effect of stormwater treatment on the number of neuromasts on the trunk (lateral body surfaces) (F_3,46_ = 8.33, p = 0.0002), with control fish averaging 92.1 neuromasts and treated fish averaging 86.6, 80, and 73.1 neuromasts, respectively, for the 50% stormwater, 100% stormwater, and 100% filtered stormwater groups. However, this effect was only significant for the fish reared in filtered stormwater (Fig. 8c, p < 0.0001).

There was a significant effect of stormwater treatment on fish size, with fish developing in stormwater being slightly but significantly smaller than fish developing in clean water or filtered stormwater (1-way ANOVA, F_3,46_ = 14.72, p < 0.001). We therefore examined the interaction between treatment and fish size (total length) on neuromast number in embryonic coho. There was no significant interaction between treatment and fish length, suggesting that observed differences in neuromast number are an effect of stormwater treatment rather than of size (2-way ANOVA, F_6,50_ = 0.646, p = 0.69).

### Water Chemistry

Given the variability in lateral line toxicity among water samples from different storms, water chemistry was measured for runoff collected from the storm events on June 2014 and January 2016 as part of other studies. Concentrations of solids and dissolved metals in 100% runoff collected on these dates (Table 1) were similar to those previously published for runoff from this collection site^5,43^. Bioretention filtration of runoff from the 2016 event resulted in increased salts and reduced solids, ammonia, copper, and zinc (Table 1). These changes are similar to a previous study reporting effectiveness of bioretention for runoff from this collection site^5,43^.

**Table 1:** Water chemistry measured in stormwater runoff samples from two collection dates. Metals are dissolved (water passed through a 0.45 μm filter). Filtered runoff had been passed through a bioretention treatment system as described in the Methods section.

Parameter	Method^c^	Detection Limit	Units	Jun 16 2014^a^	Jan 14 2016^b^	
				100% Runoff	100% Runoff	100% Filtered Runoff
pH	SM 4500HB	0.1	none	6.5 (0.1)	6.9	6.6
TSS^d^	SM 2540D	1	mg/L	52 (4)	180	12
SSC^e^	ASTM D3977–97	1	mg/L	43 (3)	220	15
DOC^f^	SM 5310B	0.5	mg/L	13 (0)	5.1	6.9
Alkalinity	SM 2320B	1	mg/L^g^	16 (0)	40	26
Hardness	EPA 200.7 calc	1	mg/L^g^	28 (1)	44	920
Ca	EPA 200.7	0.05	mg/L	9.83 (0.15)	12	230
Mg	EPA 200.7	0.01	mg/L	0.96 (0.03)	3.3	85
Ammonia^h^	EPA 350.1	0.01	mg/L	1.3 (0.02)	1.17	0.14
Nitrate	EPA 300.0	0.025	mg/L	0.88 (0.01)	0.206	<
Ortho-P	SM 4500-PE	0.005	mg/L	0.019 (0.003)	<	0.114
Cd	EPA 200.8	0.025	μg/L	0.101 (0.012)	0.076	0.076
Cu	EPA 200.8	0.1	μg/L	33.7 (0.9)	15.6	7.06
Pb	EPA 200.8	0.05	μg/L	0.44 (0.02)	0.15	0.18
Ni	EPA 200.8	0.05	μg/L	1.22 (0.98)	2.02	4.97
Zn	EPA 200.8	0.5	μg/L	92.4 (2.5)	44.9	8.9

^a^Triplicate samples showing average (standard deviation).

^b^Unique samples (no replication).

^c^Analysis method following: SM = Standard Method; ASTM = ASTM International; EPA = U.S. Environmental Protection Agency.

^d^Total suspended solids.

^e^Suspended sediment concentration.

^f^Dissolved organic carbon.

^g^as CaCO_3_.

^h^Total ammonia nitrogen. < Used when values less than detection limit.

## Discussion

We investigated the effect that stormwater runoff has on sensory hair cells of the lateral line in larval zebrafish and coho salmon. In 5–6 dpf zebrafish larvae, acute exposure does not kill hair cells, but it does reduce uptake of the dye FM 1–43FX, a key marker of hair cell function. A previous study also did not see an effect of stormwater exposure on lateral line hair cell survival, but that study used a 3 hr incubation period, rather than the 24 hr exposure period used here^5^. Their study assessed hair cell survival with DASPEI, which is not as sensitive to changes in function of the mechanotransduction apparatus as is FM 1–43FX. The lateral line is acutely sensitive to many aquatic toxicants, including bisphenol-A and heavy metals such as copper and cadmium, likely due to the external location of this sensory system and the relative permeability of hair cells to molecules in the surrounding environment^29,31,33,44^. Hair cells in 5 dpf zebrafish larvae exhibit adult-like sensitivity to some drugs, such as aminoglycoside antibiotics and chemotherapy agents^28,45,46^. Given that larval hair cells demonstrate responses to some toxicants that are similar to responses in adult fishes, our data suggest that hair cells in adult fish may also be negatively impacted by short-term stormwater exposure.

It is unclear how stormwater exposure reduces mechanotransduction function in lateral line hair cells. Crude oil exposure can alter intracellular calcium dynamics within fish cardiomyoctes^47,48^, and in hair cells, mechanosensitivity is tightly regulated by intracellular calcium levels in the hair bundle^49–51^. Therefore, the reduction in uptake of FM 1–43FX could be due to increased intracellular calcium, which could directly reduce dye uptake by closing mechanotransduction channels. In cardiomyoctes, however, crude oil exposure reduces intracellular calcium^47,48^, which would increase hair cell permeability to toxicants and facilitate hair cell damage. While PAHs can directly block some ion channels^47,48^, it is unlikely that FM 1–43FX uptake was reduced because of a direct channel block, as our experiments were performed after the fish were removed from stormwater. Altered calcium dynamics were observed in lateral line hair cells treated with aminoglycoside antibiotics and inhibition of calcium mobility prevented hair cell death from these drugs^52,53^. Therefore, stormwater exposure may disrupt intracellular calcium mobility, leading to hair cell damage. Future studies are needed to explore this hypothesis and determine the molecular signaling cascades involved.

In addition to acutely toxic effects on the lateral line, stormwater exposure during embryogenesis caused lateral line defects in both zebrafish and coho salmon. Direct comparisons between zebrafish and coho salmon data are not possible due to differences in the developmental stage at which each species was treated, the overall developmental timeline (coho salmon develop much more slowly), and experimental factors such as the stormwater exposure paradigm used here. However, these data suggest that stormwater exposure is likely to cause defects in lateral line development in a variety of fish species. This developmental effect was highly variable, with 10% stormwater from some storm events causing a reduction in neuromasts and hair cells per neuromast, while for other storm events a similar effect was only observed with 100% stormwater exposure. The concentration of PAHs and other toxicants varies with each storm, and storms following a dry period usually cause more toxic runoff than storms in the middle of the rainy season, as toxicants build up on impervious roadways during dry spells^3,4^. Stormwater with higher PAH concentrations causes more severe cardiac phenotypes^13^, and a similar effect is likely in the lateral line. Developmental stormwater exposure is also associated with reduced fish growth^5^, so it is possible that the lateral line defects are secondary to other developmental deficits such as small size. We consider this unlikely, as there was no interaction between size and neuromast number in stormwater-treated coho embryos. However, we cannot rule out a size effect, as fish development is more closely correlated with size than age.

While it is possible that PAHs are responsible for the observed defects, urban stormwater runoff contains a very complex mixture of contaminants^54^, including suspended particles and dissolved metals, in addition to organics such as PAHs. A recent study on runoff from the same stormwater collection site used for our study detected, through high resolution quadrupole time-of-flight mass spectrometry, hundreds to thousands of organic compounds - the vast majority of which remain unidentified and uncharacterized^55^. Although several metals (*e.g*., copper) are classically known to be neurotoxic to peripheral sensory systems^32,56^, toxicity is mediated by the presence of metal-scavenging organic matter (typically measured as dissolved organic carbon)^57,58^. Furthermore, metals not measured in the current study (*e.g*., cobalt) can also be toxic to the fish lateral line^59^, as can low pH and organic contaminants including pesticides and surfactants^60^.

Therefore, measuring a short list of contaminants present in urban runoff does not guarantee an ability to explain relative differences in toxicity among samples. For example, there were higher concentrations of the neurotoxic metal copper in the June 2014 sample compared to the January 2016 sample, and yet the January sample caused a stronger reduction of hair cells in zebrafish head neuromasts (64%) compared to the June 2014 sample (23%). Lower relative DOC concentrations in the January sample may help explain the difference in toxicity between the two samples, but robust analysis of water chemistry is not productive without a better understanding of the relative toxic potencies of the many different chemicals in the mixture. Similarly, the beneficial effect of bioretention filtration on zebrafish lateral line development observed for the January sample may have as much to do with what is added to the water (*e.g*., DOC, salts; Table 1) as what is removed (*e.g*., neurotoxic metals). Future studies are needed to determine the precise water constituents that caused the lateral line defects.

In zebrafish, lateral line defects were rescued by activation of Wnt signaling. Wnt controls several facets of lateral line development, including neuromast deposition along the trunk, proliferation of precursor cells, and hair cell addition^39–42^. PAHs can dysregulate ß-catenin, a key component of the Wnt pathway, leading to patterning defects in zebrafish and Pacific herring^52^. Wnt signaling is important for early cardiac development, and cardiac dysfunction is a major outcome of PAH toxicity^14,61,62^. It is unclear if the cardiac defects in PAH-exposed embryos are partially due to disrupted Wnt signaling; several studies demonstrate that PAHs act through the aryl hydrocarbon receptor (AHR) to cause cardiotoxicity^15,61^. AHR activation may cause cardiotoxicity in zebrafish by downstream disruption of Wnt signaling, suggesting that Wnt could play a central role in developmental stormwater toxicity^62^. However, as Wnt activation leads to excess hair cells in untreated zebrafish, it is possible that stormwater does not inhibit Wnt signaling, but instead that stormwater acts through a separate pathway for which Wnt can compensate. One candidate is retinoic acid signaling, which is involved in lateral line development and disrupted by PAH exposure^63,64^. Future experiments are needed to determine the signaling pathway(s) responsible for stormwater-mediated lateral line defects.

Stormwater exposure during embryonic and larval development caused a reduction in the number of trunk neuromasts, at least for some storm events, and a reduction in the number of hair cells per neuromast. Both phenotypes could occur by stormwater-mediated attenuation of cell proliferation in the developing lateral line. However, we did not find a difference in the number of BrdU-labeled hair cells in stormwater-treated larvae. These data imply that stormwater exposure does not alter proliferation. We incubated fish in BrdU from 32.5–34 hpf, a window of high proliferation during lateral line primordium migration^40,65^. However, our BrdU pulse was restricted in time to avoid embryo toxicity, so we may have missed observing an effect on progenitor proliferation. Hair cell regeneration proceeded normally in stormwater-treated larvae that were exposed to the hair cell toxicant neomycin, further evidence that stormwater does not affect cell proliferation in the lateral line. Therefore, the developmental effect likely results from a non-proliferative mechanism, such as progenitor cell survival.

We did not detect lateral line defects in zebrafish embryos raised in filtered stormwater, while filtration did not rescue lateral line morphology in coho salmon embryos. This may be due to differential sensitivity to trace levels of toxicants between the species, or to the different exposure paradigms for each species; zebrafish were exposed continuously for 4 days, while coho embryos were exposed in a pulsed manner for 43 or 71 days. Zebrafish are warm-water fishes that exhibit rapid development, while Pacific salmon are cold-water fishes with longer developmental trajectories. It is therefore unsurprising that coho embryos may be more sensitive to trace amounts of toxicants. Previous work in coho salmon shows that stormwater treatment with bioretention columns completely amelioriates overall mortality, and filtered stormwater reduces several markers of toxicity in zebrafish, including cardiac edema and fish size^5,11^. Bioretention treatment reduces the severity of micropthalmia (small eyes) but does not completely prevent this phenotype, similar to our finding that bioretention does not prevent lateral line defects in developing coho salmon. Bioretention filtration is highly effective at removing the bulk of the developmental toxicants from stormwater runoff, but sensory systems such as the eye and lateral line appear particularly susceptible to residual toxicants that remain after filtration.

In conclusion, our data demonstrate that either acute or developmental stormwater exposure can have negative consequences for the mechanosensory lateral line of zebrafish and coho salmon. While mortality and cardiotoxicity are certainly the greatest concerns, our data add to the growing body of evidence suggesting that sublethal stormwater exposure can have detrimental effects on fishes. Stormwater-related lateral line deficits have the potential to reduce prey capture and predator avoidance behaviors, increasing the risk for fish mortality.

## Methods

### Animals

All procedures were approved by the Institutional Animal Care and Use Committee at Washington State University. All experiments were performed in accordance with the relevant guidelines and regulations of Washington State University and the American Veterinary Medical Association.

#### Zebrafish

We used 5–6 days post-fertilization (dpf) zebrafish (*Danio rerio*) for acute toxicity experiments because their hair cells exhibit adult-like sensitivity to known hair cell toxicants^45,46^. Developmental studies were initiated shortly after the eggs were fertilized (4–24 hours post-fertilization (hpf)). *AB wildtype or Brn3C:mGFP transgenic fish were used for all experiments. Hair cells of Brn3c:mGFP animals express membrane-bound green fluorescent protein (GFP) in all hair cells and the transgene is turned on early in hair cell development, making this fish line ideal for developmental studies^66^.

#### Coho Salmon

Embryonic coho salmon (*Oncorhynchus kisutch*) were obtained from Grovers Creek Salmon Hatchery in Poulsbo, Washington. As part of a separate study, eggs fertilized on 23 November 2015 were reared in flow-through heath stacks with well water during development and episodically exposed to recirculating stormwater runoff treatments. Fish were collected from the hatchery on 5 January 2016 and 2 February 2016 and transported to Washington State University Vancouver for lateral line assessment.

### Stormwater collection and filtration

Stormwater runoff was collected from SR 520, a floating bridge that crosses Lake Washington in Seattle, WA as described previously^5^. Briefly, downspouts from an on-ramp to SR520 directed runoff during rain events into a stainless steel IBC tote. For studies with zebrafish, runoff was frozen in amber glass bottles and stored at −20 °C. For developmental tests with coho salmon, runoff was transported within 24 h to the hatchery where it was dispensed to stainless steel sumps for recirculting exposure to embryos. The filtered runoff treatment used bioretention cells. Cells were constructed in 55-gallon polyethylene drums fitted with a slotted PVC underdrain embedded in 30.5 cm of gravel. The well-mixed bioretention soil media (BSM) overlying the gravel (61-cm) was composed of 60% sand and 40% compost by volume as per the Western Washington Stormwater Management Manual^67^. The BSM was overlain by 2” of bark mulch. Runoff was treated at 2 L/min and transported to the filtered runoff sump in high density polyethylene buckets. Dilutions of runoff used the same water source as that used for rearing embryos between stormwater exposures. Analytical chemistry of the June 2014 and January 2016 stormwater samples was conducted as previously described^5,43^.

### Stormwater exposure

#### Zebrafish

For acute exposure, 5–6 dpf zebrafish were incubated in varying concentrations of thawed stormwater for 1–24 hr, with E2 embryo medium^68^ (EM) used to dilute stormwater to the desired concentration (1 mM MgSO_4_, 0.15 mM KH_2_PO_4_, 1 mM CaCl_2_, 0.5 mM KCl, 15 mM NaCl, 0.05 mM Na_2_HPO, and 0.7 mM NaHCO_3_ in dH_2_O, pH 7.2). After exposure, fish were rinsed twice in fresh EM, and hair cells were assessed as described below. For hair cell regeneration studies, 5 dpf zebrafish were treated for 1 hr with 300 µM neomycin, a known hair cell toxicant^28^. Fish were then rinsed in fresh EM and allowed to recover for 24 or 48 hr prior to hair cell assessment. In either set of experiments, control fish were treated with EM only (no stormwater).

For developmental studies, zebrafish were exposed to varying dilutions of thawed stormwater (diluted with fish facility water), beginning at 4–24 hpf and ending at 3–4 dpf. Stormwater was refreshed daily. For a subset of these studies, the Wnt activator lithium chloride was added as a cotreatment with stormwater^42^. 4–6 hpf Brn3c:mGFP zebrafish were exposed to 15 mM LiCl with or without stormwater, again with solutions refreshed daily. All experiments were conducted with water from multiple storm events; in some cases, only one representative result is shown for experiments where the results were consistent across storm events. In pilot tests with zebrafish, freezing runoff did not affect its developmental toxicity^5^. Control fish were treated with fish facility water only; this water has lower conductivity than EM and is preferable for developmental studies (Coffin, unpublished observation).

#### Coho salmon

For developmental studies in coho salmon, embryonic coho were episodically exposed for 24–48 hr to either 50% or 100% stormwater, or with 100% stormwater that was first treated in bioretention columns as described above. At the end of the exposure, the water source was switched back to flow-through with well water. Control fish were exposed to well water only. Runoff tended to be slightly cooler than the well water used for rearing between runoff exposures, such that there was a slight difference in degree days of the embryos across treatments. On 5 January (43 dpf), embryos had been exposed to 10 episodic runoff exposures, totaling 19 days of development. Their degree-days (accumulated thermal units, in degree Celcius) were 352 for controls, 345 for 50% runoff treatment, 338 for 100% runoff, and 337 for 100% filtered runoff. On 2 February (71 dpf) embryos had been exposed to an additional 5 runoff events, for a total exposure of 29 days.

### Lateral line assessment

#### Vital dye labeling

Neuromasts in live, anesthetized fish were assessed using the mitochondrial dye 2-(4-(dimethylamino)styryl)-N-Ethylpyridinium iodide (DASPEI, Life Technologies, Carlsbad, CA, USA), which specifically labels lateral line hair cells and olfactory receptors^28,37^. Zebrafish larvae were incubated in EM containing 0.005% DASPEI for 15 min, rinsed twice with fresh EM, then anesthetized by immersion in buffered 0.001% MS-222 in fresh EM (3-aminobenzoic acid ethyl ester methanesulfonate, Argent Labs, Redmond, WA, USA). Neuromasts were viewed with a Leica M165F fluorescent stereomicroscope (Leica Microsystems, Buffalo Grove, IL, USA). We assessed the same 10 anterior neuromasts for each fish^23^ (SO1, SO2, IO1-IO4, M2, MI1, MI2, O2, see Fig. 1), with each neuromast scored as 0 (no labeling), 1 (moderate labeling), or 2 (bright labeling). Scores for each neuromast were summed, resulting in a total score of 0–20 per fish^28^. As neuromast locations are stereotyped in zebrafish larvae, absence of a neuromast is likely a treatment effect rather than developmental variation. While hair cell assessment was not performed blind, we have previously performed side-by-side comparisons of blinded vs. unblinded DASPEI assessment and found no differences in scoring^69^. The reader is referred to Raible and Kruse (2000) for details on zebrafish neuromast nomenclature^23^.

For embryonic coho salmon, fish were manually dechorionated and incubated with DASPEI as described above, except that we used a 20 min incubation period, which better labels the lateral line in salmonids, including the canal neuromasts^70^. We then counted the number of neuromasts in four head regions: the infraorbital canal, the preopercular canal, the stitch of opercular superficial neuromasts, and the y-shaped stitch of superficial neuromasts on the dorsal surface of the head^70,71^. Details of salmonid lateral line morphology are shown in Brown *et al*. (2013)^70^. We also quantified the number of neuromasts on the trunk. Counts from the four head regions were summed to obtain one head value per fish. Coho embryos were evaluated blind to treatment.

#### Hair cells counts

Hair cell counts were conducted in Brn3c:mGPF zebrafish larvae, where all hair cells are GFP+. Fish were rinsed in fresh EM, anesthetized with buffered 0.001% MS-222, and mounted on bridged coverslips. Fish were visualized with a Leica DMI4000 B compound fluorescent microscope or Leica SP8 confocal microscope. Hair cells in neuromasts IO1-IO3, M2, and OP1 were quantified and summed to arrive at one value for each fish (see Fig. 1). We also examined multiple trunk neuromasts. For the June 2014 storm we quantified hair cells on the two most posterior neuromasts on the tail, which exhibited considerable variation even in control animals. Therefore, we elected to focus on the head region and the anterior-most neuromasts of the trunk for additional experiments.

#### FM 1–43

To examine the relative health of surviving hair cells, we used the vital dye FM 1–43FX, which enters hair cells through the mechanotransduction channels and is considered a proxy for hair cell viability^34,35^. Fish were exposed to 3 µM FM 1–43FX (Life Technologies) for 30 sec. Fish were then rinsed 3 times in fresh EM, euthanized with an overdose of buffered MS-222, and fixed in 4% paraformaldehyde (PFA) overnight at 4 °C. FM 1–43FX-labeled fish were imaged with a Leica SP8 confocal microscope, and relative fluorescent intensity was quantified from defined regions of interest using ImageJ^72^.

### Cell proliferation assay

At 32.5–34 hpf, Brn3c:mGFP zebrafish embryos were dechorionated, then exposed to 10 mM 5-bromo-2-deoxyuridine (BrdU, Sigma-Aldrich, St. Louis, MO, USA), supplemented with 15% dimethylsulfoxide (DMSO) in EM^65^. BrdU treatment was conducted for 90 min; 30 min at 4 °C, followed by 60 min at 28 °C. Embryos were rinsed in fresh EM, then transferred to fish room water and allowed to develop to 4 dpf. The exposure window of 32.5–34 hpf is based on previous studies showing substantial levels of proliferation in lateral line precursors during this developmental period^40,65^.

At 4 dpf, zebrafish were processed for BrdU detection modified from published protocols^28^. All compounds were obtained from Sigma-Aldrich unless specified, and all steps were performed at room temperature unless noted. Embryos were euthanized with buffered MS-222 and fixed for 1 hr in 4% PFA. Tissue was permeabilized in three rinses of PBDT (1% DMSO and 0.5% Triton-X 100 in phosphate buffered saline (PBS)) at 20 min increments, incubated in purified water for 30 min, and blocked in antibody block solution (5% normal goat serum (NGS) in PBDT) for 1 hr. Tissue was then incubated in polyclonal anti-GFP primary antibody (Life Technologies), diluted 1:200 in PBDT with 1% NGS, applied overnight at 4 °C. Tissue was rinsed in PBDT prior to 5 hr of secondary antibody exposure (Alexa Fluor 488 goat anti-rabbit IgG in PBDT, 1:500, Life Technologies). The fish were refixed with PFA for 20 minutes and placed in methanol for 1 hour at −20 °C. After rehydrating in a graded methanol series, fish were rinsed with PBDT and digested with Proteinase K (10 ug/mL in PBDT) for 20 min. Tissue was rinsed with PBDT, refixed with PFA for 20 min, rinsed again, and exposed to 2 N HCl for 1 hr. Fish were rinsed again, blocked in 10% NGS in PBDT for 1 hr, and incubated overnight at 4 °C in monoclonal anti-BrdU antibody diluted 1:100 in PBDT with 1% NGS. Fish were then rinsed in PBDT and exposed to secondary antibody (Alexa Fluor 568 goat anti-mouse IgG in PBDT, 1:200, Life Technologies) for 5 hr. Fish were again rinsed and stored in 50% PBS:glycerol for imaging on a Leica SP8 confocal microscope.

BrdU-positive hair cells represent hair cells that resulted from proliferation of precursor cells. These cells were defined as cells that were positive for BrdU (which labels all proliferating cells), but also for GFP, as all hair cells in the Brn3c:mGFP transgenic line express GFP. BrdU + hair cells were quantified in 5 head neuromasts (IO1- IO3, M2, and OP1) and in the two most anterior trunk neuromasts. Numbers were summed to achieve one value per fish.

### Statistical analysis

Data were analyzed using a 2-tailed t-test or one- or two-way ANOVA, as appropriate, with Prism v. 7 or SPSS Statistics. Data are presented as mean + 1 s.d.

### Data availability

The datasets generated during and/or analysed during the current study are available from the corresponding author on reasonable request.
